# Endothelin-1 Single Nucleotide Polymorphisms and Risk of Pulmonary Metastatic Osteosarcoma

**DOI:** 10.1371/journal.pone.0073349

**Published:** 2013-09-12

**Authors:** Xiaofang Zang, Yong Zhou, Zufa Huang, Chaoyue Zhang

**Affiliations:** Department of Orthopaedics, the Third Xiangya Hospital, Central South University, Changsha, Hunan, China; Universitat Pompeu Fabra, Spain

## Abstract

Pulmonary metastases are the major cause of death of osteosarcoma (OS) patients. Endothelin-1 (ET-1) reportedly plays an important role in OS metastasis. In the present study, we for the first time explored the association of *ET-1* SNPs with the risk of pulmonary metastatic OS. We genotyped three SNPs (rs1800541, rs2070699 and rs5370) in the *ET-1* gene in a case-control study, using 260 pairs of age-, sex-, residence area- and tumor location-matched subjects. Patients with pulmonary metastatic OS and patients with localized high-grade (stage IIB) OS were enrolled as cases and controls, respectively. The G allele at rs1800541 was found associated with reduced risk of pulmonary metastatic OS after adjustment for body mass index, systolic blood pressure, diastolic blood pressure and the plasma ET-1 level (*P*=10^-4^; adjusted OR, 0.55; 95% CI, 0.42-0.70), while the G allele at rs2070699 was not significantly associated with the risk of pulmonary metastatic OS (*P*=0.15; adjusted OR, 1.15; 95% CI, 0.87-1.50). The mRNA and the secreted protein levels of ET-1 in primary OS cell cultures (POCCs) established from surgically resected primary OS in the rs1800541 TT homozygotes were higher than those from the TG heterozygotes (*P*<0.05), who in turn showed higher ET-1 mRNA and secreted ET-1 levels than the GG homozygotes (*P*<0.05). In the control subjects, the rs1800541 TT homozygotes showed an 18.4% relapse rate, significantly higher than that of the GG homozygotes (0%) (*P*<0.01). On the other hand, the GG homozygotes showed a 71.4% complete recovery rate, significantly higher than that of the TG heterozygotes (7.3%) and the TT homozygotes (0%) (*P*<0.01). This study provides the first evidence of an association between the *ET-1* gene SNPs and the risk of pulmonary metastatic OS.

## Introduction

Osteosarcoma (OS) is the most frequent malignant bone tumor in children and adolescents [1]. OS is a devastating disease, characterized by high local aggressiveness and a tendency to metastasize to the lungs and distant bones. In spite of the use of neoadjuvant chemotherapy and improvement in surgical technology that have increased the survival rate to 65-75%, pulmonary metastasis occurs in approximately 40%-50% of OS patients and remains a major cause of fatal outcome [2-4]. The cure rate of OS is approximately 65% for patients with localized diseases. When presenting with metastases at the time of diagnosis, the survival rate is 25% [5,6]. Thus, it is important to uncover the molecular mechanisms involved in OS progression, particularly, pulmonary metastasis. Although there have been many studies on its genetics, biology, pathology and clinical aspects, the etiology of osteosarcoma is not well understood. Previous studies suggest a genetic predisposition of osteosarcoma [7].

Endothelin-1 (ET-1) is a potent vasoconstrictor initially isolated from endothelial cells [8]. ET-1 signaling reportedly is involved in a wide range of cancer-relevant processes, such as inhibition of apoptosis, matrix remodeling, bone deposition, and metastases [8]. ET-1 and ET A receptor (ETAR) are expressed in OS tissue and cells [8,9]. Previous studies suggest that ET-1 is important for OS progression and metastasis [8-10]. Zhao et al. reported that ET-1 could promote OS cell invasion and survival [8]. Felx et al. reported that ET-1 could promote metalloproteinase induction in human OS [9]. Li et al. showed that ETAR, the major target for ET-1, was critical for OS pulmonary metastasis in an orthotopic xenograft OS model [10]. Single nucleotide polymorphisms (SNPs) of the *ET-1* gene have been reportedly associated with pulmonary and cardiovascular diseases [11-14]. Despite the important role of ET-1 signaling in OS progression, no study has investigated the association of *ET-1* gene polymorphisms with OS. In the present study, we for the first time explored the association of *ET-1* SNPs with the risk of pulmonary metastatic OS in a case-control study, using 260 pairs of age-, sex-, residence area- and tumor location-matched subjects.

## Materials and Methods

### Ethics Statement

This study was approved by the Ethics Committee of the Third Xiangya Hospital, Central South University. Written informed consent was obtained from adult participants or the parent or guardian of minor participants before the start of the study.

### Subjects

From January 2007 to July 2012, blood samples were collected from 260 Han Chinese patients with pulmonary metastatic (stage III) OS at the Third Xiangya Hospital of Central South University. 260 age-, sex-, residence area- and tumor location-matched Han Chinese patients diagnosed with stage IIB OS (localized high-grade OS with extracompartmental lesions) were recruited as controls [15]. All diagnoses were based on biopsy. The inclusion criteria were as follows: (1) metastatic pulmonary OS (for cases) or stage IIB OS (for controls) at diagnosis; (2) had not received any treatment; (3) without a family history of osteosarcoma or any other cancers. Patients with any other malignancies were excluded. Baseline characteristics of all subjects are summarized in Table 1. After blood sample collection, all subjects received neoadjuvant chemotherapy followed by surgical resection of the primary tumor.

**Table 1:** Characteristics of study subjects.

Characteristics		Cases (n=260)	Controls (n=260)	*P*
			
Age (years)		16.5 ± 8.3	16.8 ± 7.9	0.67
Age Range (years)		4-37	5-34	N/A
Age Group n(%)				
≤20 years		190 (73.1)	190 (73.1)	
>20 years		70 (26.9)	70 (26.9)	1.00
Gender n(%)				
Male		155 (59.6)	155 (59.6)	
female		105 (40.4)	105 (40.4)	1.00
Body Mass Index (kg/m^2^)		17.8 ± 3.2	18.3 ± 3.5	0.09
Tumor Location				
Long Tubular bones		196 (75.4)	196 (75.4)	
Axial skeleton		64 (24.6)	64 (24.6)	1.00
Systolic Blood Pressure (mmHg)		112.5 ± 14.3	110.7 ± 16.5	0.18
Diastolic Blood Pressure (mmHg)		76.7 ± 5.2	76.1 ± 5.9	0.21
Plasma ET-1 Level (pg/mL)		14.3 ± 1.9	14.1 ± 1.5	0.19

Note: For continuous variables, all values were expressed as Mean±SD. Independent student t tests were performed to compare means between the groups. For categorical variables, all values were expressed as n(%) and comparisons were performed with Chi-square tests.

### SNP Selection and Genotyping

Three SNPs in the *ET-1* gene, including rs1800541 in the promoter region, rs2070699 in intron, and rs5370 in the coding region were selected. All three SNPs had been involved in multiple other studies [11-14]. Genomic DNA was isolated from white blood cells using the phenol/chloroform method and was stored in 400 ml of TE (10 mM Tris/HCl and 1 mM EDTA (pH 8.0). As previously described, SNPs were genotyped using SNPlex assays (Applied Biosystems, Foster City, CA, USA) based on oligonucleotide ligation assay for capillary electrophoresis on ABI 3700 DNA Analyzers (Applied Biosystems) [16,17]. Quality control was performed by sequencing all three SNPs in 120 subjects randomly selected from the control group. The discrepancy rate was 1.7%.

### Primary OS Cell Culture (POCC)

POCC were obtained as previously described [8]. Briefly, immediately after excision, the osteosarcoma specimens were mechanically minced and digested with 0.13% collagenase (Sigma), 375 U/ml DNAse (Sigma), and 0.1% hyaluronidase (Sigma). The cell suspension was passed through a mesh of 200-µm width and cultured in RPMI 1640 (Invitrogen, Carlsbad, CA, USA) supplemented with 10% fetal calf serum (Invitrogen) and 50 µg/ml gentamycin (Invitrogen) at 5% CO_2_ and 37°C. The culture medium was changed when the cells were at least 80% confluent. When 100% confluence was reached, the cells were passaged for future generation. At the fourth passage, part of POCC from each patient was subject to Giesma staining. Two pathologists independently examined each Giemsa-stained POCC to determine the percentage of tumor cells in the culture. 10 high-power (200×) view fields were randomly picked in each sample; tumor cells were then identified by tumor cell morphology [18] and counted against non-tumor cells by each pathologist independently. A reading within ±5% of the other for each POCC was considered as an agreement. Cohen’s kappa coefficient was calculated to show the interobserver variability (0.81-0.85 in the present study).

### Real-Time Quantitative Reverse Transcription PCR

Total RNA were prepared from 10^6^ cells from each POCC using TRIzol reagent followed by purification with TURBO DNA-free System (Ambion, Austin, TX, USA). The cDNAs were synthesized using SuperScript II reverse transcriptase (Invitrogen, Carlsbad, CA, USA). Real-time quantitative PCR was performed on an Abi-Prism 7700 Sequence Detection System, with use of the fluorescent dye SYBR Green Master Mix (Applied Biosystems) as described by the manufacturer. The results were normalized against that of the housekeeping gene glyceraldehyde-3-phosphate dehydrogenase (GAPDH) in the same sample. The primers used are as follows: for *ET-1*, 5′-TCCTCTGCTGGTTCCTGACT-3′ (forward) and 5′-CAGAAACTCCACCCCTGTGT-3′ (reverse); for *GAPDH*, 5′-GTCAGTGGTGGACCTGACCT-3′ (forward) and 5′-TGCTGTAGCCAAATTCGTTG-3′ (reverse).

### Enzyme-Linked Immunosorbent Assay (ELISA)

The cell-secreted ET-1 levels in POCC supernatants were determined using an ET-1 ELISA kit (R&D Systems, Minneapolis, MN, USA). In brief, cells were grown to confluence in 10-cm dishes in RPMI 1640 medium supplemented with 10% fetal calf serum, followed by replacing the medium with serum-free medium and further incubation for 16 hours. The cell culture supernatants were collected for ELISA according to the manufacturer’s instructions. ELISA-detected ET-1 concentrations were normalized against cell number (per 10^6^ cells).

### Statistical Analysis

Hardy-Weinberg equilibrium analysis for genotype distribution in controls was carried out by a Chi-square goodness-of-fit test. Differences in genotype and allele frequencies between cases and controls were determined using Chi-square test. Logistic regression was performed to assess OR and 95% CI. All the statistical analyses were performed with SAS 9.1.3. Values for all continuous variables are expressed as Mean±SD. The statistical significance level of this study was set at a two-tailed α=0.05.

## Results

As this was an age, sex and tumor location matched cases-control study, there was no significant difference in age, sex and distribution of tumor location between pulmonary metastatic OS cases and controls (Table 1). There was also no significant difference in body mass index (BMI), systolic blood pressure, diastolic blood pressure and plasma ET-1 levels between cases and controls (Table 1).

As shown in Table 2, among the three SNPs, rs5370 was found to deviate significantly from Hardy-Weinberg equilibrium in controls and therefore excluded from later analyses. As shown in Table 3, the G allele at rs1800541 was significantly associated with reduced risk of pulmonary metastatic OS after adjustment for BMI, systolic blood pressure, diastolic blood pressure and the plasma ET-1 level (*P*=10^-4^; adjusted OR, 0.55; 95% CI, 0.42-0.70), while the G allele at rs2070699 was not significantly associated with the risk of pulmonary metastatic OS (*P*=0.15; adjusted OR, 1.15; 95% CI, 0.87-1.50).

**Table 2:** Hardy-Weinberg Equilibrium (HWE) Test on Controls in the Study.

Reference SNP ID (rs)	HWE Test *P* Value	Chromosome Position	Region in Gene	Alleles^a^
rs1800541	0.087	12397205	Promoter	T:G
rs2070699	0.426	12400758	Intron	T:G
rs5370	0.043*	12404241	Lys>Asn	G:T

Note: *P<0.05; ^a^ Alleles are presented as major: minor allele.

**Table 3:** Genotype and Allele Frequencies of *Endothelin-1* SNPs among Pulmonary Metastatic Osteosarcoma Cases and Controls.

	Cases/Controls n(%)	Crude OR (95% CI)	*P*	Adjusted OR^a^ (95% CI)		*P*
					
rs1800541						
Genotype						
GG	7 (2.7)/28 (10.8)	0.19 (0.08-0.44)			0.32 (0.13-0.62)	
TG	69 (26.5)/96 (36.9)	0.53 (0.36-0.78)			0.69 (0.45-0.87)	
TT	184 (70.8)/136 (52.3)	1.00	10^-7^		1.00	10^-4^
Allele						
G	83 (16.0)/152 (29.2)	0.46 (0.34-0.62)	10^-7^		0.55 (0.42-0.70)	
T	437 (84.0)/368 (70.8)	1.00			1.00	10^-4^
rs2070699						
Genotype						
GG	57 (23.8)/47 (18.1)	1.63 (0.98-2.73)			1.44 (0.89-2.48)	
TG	145 (55.8)/135 (51.9)	1.44 (0.96-2.18)			1.31 (0.84-1.97)	
TT	58 (20.4)/78 (30.0)	1.00	0.12		1.00	0.22
Allele						
G	259 (49.8)/229 (44.0)	1.26 (0.99-1.61)			1.15 (0.87-1.50)	
T	261 (50.2)/291 (56.0)	1.00	0.07		1.00	0.15

Note: OR, odds ratio. ^a^ Adjusted for body mass index, systolic blood pressure, diastolic blood pressure and the plasma ET-1 level.

To determine the effects of the rs1800541 polymorphism on blood pressure and ET-1 levels, we next compared systolic blood pressure, diastolic blood pressure and plasma ET-1 levels in pulmonary metastatic OS cases and controls by the rs1800541 GG, TG, and TT genotypes. As shown in Table 4, the rs1800541 polymorphism had no significant effects on blood pressure and plasma ET-1 levels in both cases and controls. To investigate the effects of the rs1800541 polymorphism on secreted ET-1 levels from the OS cells, POCCs were established from surgically resected primary OS from the study subjects. Based on a two-tailed α=0.05, β=0.20, power=0.80, and an effect size=1.5, a sample size of 9 was originally calculated for comparison of means between two groups. Taken into account of an anticipated 40% failure rate in establishing POCC, 15 subjects were randomly selected for each of the GG genotype, the TG genotype and the TT genotype groups in cases and controls to ensure an adequate final sample size of POCC (n≥9). Giemsa staining coupled with tumor cell morphology analysis revealed that the percentage of OS tumor cells in the POCCs was about 80% in all groups without statistically significant group difference. In both pulmonary metastatic OS cases and controls, the ET-1 mRNA level and the secreted ET-1 level in POCCs established from the TT homozygotes were significantly higher than those from the TG heterozygotes (*P*<0.05), who showed higher ET-1 mRNA and secreted ET-1 levels than the GG homozygotes (*P*<0.05) (Table 4). In addition, all the genotype groups in pulmonary metastatic OS cases showed higher ET-1 mRNA and secreted ET-1 levels than their counterparts in the controls (*P*<0.05) (Table 4).

**Table 4:** Blood Pressure and Endothelin-1 (ET-1) Levels in Study Subjects by the rs1800541 Polymorphism.

	Cases (n=260)			P	Controls (n=260)			P
rs1800541 Polymorphism	GG	TG	TT		GG	TG	TT	
	(n=7)	(n=69)	(n=184)		(n=28)	(n=96)	(n=136)	
Systolic Blood Pressure (mmHg)	112.7±19.2	114.2±17.6	115.1±13.5	0.128	111.0±17.9	113.5±17.1	114.9±14.3	0.202
Diastolic Blood Pressure (mmHg)	76.1±7.3	77.5±6.6	77.9±5.2	0.252	76.3±6.7	77.4±6.3	78.1±5.4	0.309
Plasma ET-1 Level (pg/mL)	14.7±2.0	15.5±2.2	16.3±2.8	0.157	14.2±2.2	15.3±2.2	16.3±1.9	0.225
ET-1 mRNA Level in POCC	0.37±0.05	0.53±0.04^a,c^	0.78±0.03^a,b,c^	0.002*	0.20±0.03^a^	0.35±0.03^b,c,e^	0.56±0.03^a,c,d,e^	0.004*
	(n=10)	(n=11)	(n=11)		(n=9)	(n=9)	(n=10)	
Secreted ET-1 in POCC (pg/mL/10^6^ cells)	25.4±7.6	35.1±11.1^a,c^	44.8±11.2 ^a,b,c^	0.008*	16.5±6.9^a^	24.9±7.0 ^b,c,e^	36.8±10.3 ^a,c,d,e^	0.007*
	(n=10)	(n=11)	(n=11)		(n=9)	(n=9)	(n=10)	

Note: All values were expressed as Mean±SD. POCC, primary osteosarcoma cell culture. *P<0.05; ^a^ *P*<0.05 vs. GG in cases; ^b^ *P*<0.05 vs. TG in cases; ^c^ *P*<0.05 vs. GG in controls; ^d^ *P*<0.05 vs. TG in controls; ^e^ *P*<0.05 vs. TT in cases.

To determine the effects of the rs1800541 polymorphism on prognosis of patients with localized high-grade OS, we next compared the relapse rate and the complete recovery rate in control subjects by the rs1800541 GG, TG, and TT genotypes. As shown in Table 5, in the control subjects, the TT homozygotes showed an 18.4% relapse rate, significantly higher than that of the GG homozygotes (0%) (*P*<0.01). On the other hand, the GG homozygotes showed a 71.4% complete recovery rate, significantly higher than that of the TG heterozygotes (7.3%) and the TT homozygotes (0%) (*P*<0.01) (Table 5).

**Table 5:** Relapse Rate and Complete Recovery Rate in Control Subjects by the rs1800541 Polymorphism.

	Controls (n=260)			
rs1800541	GG	TG	TT	*P*
Polymorphism	(n=28)	(n=96)	(n=136)	
	n(%)	n(%)	n(%)	
Relapse	0 (0)	10 (10.4)	25 (18.4)	
				<0.01^a^
No Relapse	28 (100)	86 (89.6)	111 (81.6)	
Complete Recovery	20 (71.4)	7 (7.3)	0 (0)	
				<0.01^a,b^
Other Than Complete Recovery	8 (28.6)	89 (92.7)	136 (100)	

Note: POCC, primary osteosarcoma cell culture. ^a^ Fisher’s exact *P* value for GG vs. TT; ^b^ Fisher’s exact *P* value for TG vs. TT.

## Discussion

Pulmonary metastases are the major cause of death of OS patients [19]. ET-1 reportedly plays an important role in OS metastasis [8-10]. In the present study, we for the first time report that a SNP at polymorphic site rs1800541 in the *ET-1* gene is associated with increased risk of pulmonary metastatic OS.

The major effects of ET-1 appear to be on the cardiovascular and the pulmonary systems. All three *ET-1* gene SNPs (rs1800541, rs2070699, and rs5370) included in the present study have been involved in other studies [11-14]. Charu et al reported that the rs2070699 polymorphism was associated with high altitude pulmonary edema [11]. Zhu et al reported that the rs1800541 and the rs5370 polymorphisms were respectively associated with asthma and bronchial hyperreactivity [12]. Panoulas et al showed that rheumatoid arthritis patients carrying an rs1800541-rs5370 haplotype appeared more likely to be hypertensive [13]. Rankinen et al showed that hypertension risk associated with rs5370 was particularly enhanced by an rs2070699 polymorphism [14]. The previous findings indicate that *ET-1* gene SNPs rs1800541, rs2070699 and rs5370 are associated with pulmonary and cardiovascular diseases in an inter-related way, which suggests that the SNPs may significantly alter the ET-1 effects on the cardiovascular and the pulmonary systems. This was the basis of our selection of the SNPs in this study.

We found that the G allele at rs1800541 was significantly associated with reduced risk of pulmonary metastatic OS even after adjustment for BMI, systolic blood pressure, diastolic blood pressure and the plasma ET-1 level. In other words, the T allele at rs1800541 was significantly associated with high risk of pulmonary metastatic OS. The rs1800541 polymorphism showed no significant effects on blood pressure and plasma ET-1 levels in both cases and controls, suggesting that the plasma ET-1 level and the ET-1 effects on blood pressure are not linked with OS progression from localized diseases to pulmonary metastases. This explains why the association of the rs1800541 polymorphism with the risk of pulmonary metastatic OS remained significant even after adjustment for blood pressure and the plasma ET-1 level. The potential functional link between the rs1800541 polymorphism with increased risk of pulmonary metastatic OS was found at the mRNA and the secreted protein levels of ET-1 in POCC derived from surgically resected primary OS in the study subjects. The ET-1 expression levels in POCCs from the rs1800541 TT homozygotes were significantly higher than those from the TG heterozygotes, who showed higher ET-1 expression levels than the GG homozygotes. This suggests that the elevated local ET-1 levels promoted by gene dosage effect of the T allele at rs1800541 in the primary OS may account for increased risk of OS pulmonary metastases, which is supported by previous in vitro studies [8,9]. The rs1800541 GG, TG, and TT genotype groups in pulmonary metastatic OS cases showed higher ET-1 expression levels than their counterparts in the controls, suggesting that other factors besides the T allele may further enhance ET-1 expression in pulmonary metastatic OS. In addition, a pervious study suggests that although it is known by many studies that elevated ET levels can be detected in the plasma as a result of overproduction of ET released from pathologic tissues, systemic circulation may not entirely reflect local changes in the bones [20]. Our findings support this notion.

The strength of the present study is that we used a relatively large sample size of untreated subjects with stage IIB or pulmonary metastatic OS in a matched case-control study (n=520 in total; case, n=260; control, n=260) with adjustment for multiple relevant factors, which was aimed to generate reliable results. Patients with stage IIB OS (localized high-grade OS with extracompartmental lesions) were selected as controls for metastatic pulmonary OS cases. For both cases and controls, only subjects who had received no treatment at the beginning of the study were enrolled to avoid possible alterations of ET-1 levels by treatment. The advantage of using stage IIB OS patients as controls in the present study is that we could identify genetic factors immediately critical for OS progression from localized diseases to pulmonary/distant metastases. The deficiency of this design is that since the diagnoses were made at the beginning of the study, some controls (stage IIB OS) may soon develop into cases (pulmonary metastatic OS), which would likely obscure the effects of genetic factors due to mixing potential cases with controls. To make up for the deficiency, we subsequently followed up the relapse rate and the complete recovery rate by the rs1800541 GG, TG, and TT genotypes in the control subjects. The TT homozygotes showed a significantly higher relapse rate than that of the GG homozygotes. On the other hand, the GG homozygotes showed a significantly higher complete recovery rate than that of the TG heterozygotes and the GG homozygotes. The results indicate that both the T and the G alleles have a gene dosage effect on the prognosis of patients with localized high-grade OS, only in opposite directions. Since the findings were based on data collected until submission of the present manuscript, this observational study is still ongoing and expected to yield more significant results over time.
